# A participatory evaluation of legal support in the context of health‐focused peer advocacy with people who are homeless in London, UK

**DOI:** 10.1111/hsc.14111

**Published:** 2022-12-05

**Authors:** Andy Guise, Martin Burrows, Adam Marshall

**Affiliations:** ^1^ King's College London London UK; ^2^ Groundswell London UK

**Keywords:** action research, homelessness, law, participative research

## Abstract

Legal problems can be cause and consequence of ill‐health and homelessness, necessitating efforts to integrate responses to these challenges. How to respond to legal issues within the context of health services for people who are homeless is though unclear. Groundswell piloted providing legal support to peer advocates (who have current or past experience of homelessness) and clients currently homeless in addition to their health‐focused work. A participatory action‐research design evaluated the emerging programme. Groundswell staff, both researchers and those involved in service delivery, co‐led the research alongside an external researcher. Qualitative methods were used to understand the experiences of legal support. We interviewed peer advocates and volunteers (*n* = 8), Groundswell clients (*n* = 3) and sector stakeholders (*n* = 3). Interviews were linked to regular reflective recorded meetings (*n* = 7) where Groundswell staff and researchers discussed the programme and the evaluation. Data were analysed thematically. The findings focus on three themes. First, peer advocates' and clients' legal needs involve an experience of being overwhelmed by system complexity. Second, the legal support to peer advocates aided in brokering and signposting to other legal support, in the context of a supportive organisational culture. Third, support to clients can be effective, although the complexity of legal need undermines potential for sustainable responses. In conclusion, legal support for peer advocates should be developed by Groundswell and considered by other similar agencies. Legal support to people who are currently street homeless requires significant resources and so health‐focused third‐sector organisations maybe unable to offer effective support. Other modes of integration should be pursued. Findings also have implications for how the third sector relates to the government agencies implicated in the legal challenges facing people who are homeless.

**What is known about this topic?**

- The health of people who are homeless in the UK and other settings is often bound up with legal problems.
- How to address legal issues in the context of health service delivery for people who are homeless is unclear.
- Even less is known about the delivery of legal support in contexts of peer‐led services.

**What this paper adds?**

- Legal support comes in the context and experience of the ‘overwhelming complexity’ of legal needs and systems.
- Legal support to peer advocates who provide health services is feasible, within the context of a supportive organisational culture.
- Legal support to people currently homeless faces many challenges to sustainability.

## 1 INTRODUCTION

### 1.1 Legal need and support for people experiencing homelessness

Homelessness is increasingly recognised as a growing crisis across high‐income settings in Europe and North America, with implications for the delivery of health and social care and support (Fazel et al., 2014; Liu & Hwang, 2021). Over 200,000 people in England—where this study is focused—were experiencing severe forms of homelessness in 2019 (whether rough sleeping [i.e. unsheltered/sleeping on the streets], or in short‐term or unsuitable accommodation; Fitzpatrick et al., 2021). Homelessness, in its various forms, is often linked to substance use, poor mental health, prison and sex work (Aldridge et al., 2017a), and is in turn linked to living with a range of chronic and acute health conditions (Aldridge et al., 2017b; Rogans‐Watson et al., 2020).

Experiences of homelessness and ill‐health can emerge from long‐term histories of poverty, abuse, housing, unemployment and legal need or problems with criminal authorities (Fitzpatrick et al., 2011). In settings like the UK, there are social welfare programmes available that can prevent homelessness or provide support to people who are homeless, and health care is freely available, although these legal entitlements can be bounded or undermined, for example by models of welfare delivery that are experienced as stigmatising, shortages of housing or policies that limit entitlements by immigration status (Crisis, 2021; Gallent, 2019; Thompson et al., 2020). Such UK entitlements and services contrast in varying ways with other high‐income settings: some European settings can be seen to have more comprehensive support to address homelessness (Kaakinen & Turunen, 2021), whilst other settings—notably the US—frequently lack comprehensive housing or health care support that could prevent homelessness or support people who are currently homeless (Hopper, 2003).

Considerable evidence exists across settings for homelessness and poor health being associated with legal problems (Balmer et al., 2006; Buck et al., 2005). Studies across contexts have reported how people who are homeless can experience victimisation by legal authorities, are victims of crime, can be involved in illegal activities, and can be engaged in the justice system and yet have limited access to legal support (Fitzpatrick et al., 2012; Gibson, 2011; Hall, 2017; Moran & Atheron, 2019). Crucially, such experiences can have multiple pathways, with homelessness interacting in multiple ways with, for example, drug use, prison and other experiences (Boyd et al., 2016).

There is a lack of evidence for interventions or policies that could address the linked legal and health needs for people who are homeless (Luchenski et al., 2017). Welfare and legal advice have been provided in primary care settings (Moffatt et al., 2004; Woodhead et al., 2017), although with limited evidence for its impact on health outcomes (Abbott & Hobby, 2000). Recent reviews have though sought to develop theory in this area (Allmark et al., 2013). Further research is needed to understand potential for legal support in the context of health care with people who are homeless.

### 1.2 Groundswell's progression programme and the piloting of legal support

Groundswell is a charity based in London, UK that works with people with experience of homelessness. They deliver a range of projects which put people who are homeless at the heart of solutions to homelessness: focusing on service user participation in policy, service delivery and peer research. Their Homeless Health Peer Advocacy service, delivered by Peer advocates—who themselves have past or current experiences of homelessness—support people to attend health care appointments. Peer advocates may themselves have legal need and are working with clients who may also have legal need.

Each of Groundswell's peer advocates accesses a ‘progression programme’ that aims to support people towards employment, through providing person‐centred support and coaching (Finlayson et al., 2016). This progression programme includes one‐to‐one support from progression advisers, group clinical supervision, sessions with an employment coach, a progression bursary and a bespoke training programme. A team of two progression advisors within Groundswell coordinate progression support and deliver coaching sessions to help all peer advocates, volunteers and staff with experience of homelessness identify their goals and overcome the barriers that may be preventing them from achieving them. Many of Groundswell's volunteers have gone on to jobs including nurses, carers, interpreters or support workers within health and social care provision.

In 2018 Groundswell piloted enhanced support within the ‘progression programme’ to respond to the legal needs of peer advocates and clients. Legal needs here is understood broadly, to include not just interactions with justice and legal systems, but also engaging with official bodies and authorities where legal rights and entitlements are involved. Legal needs had already been identified as an issue for Peer Advocates, yet Groundswell were prompted to develop their own response owing to a reduction in support elsewhere following legal aid cuts under UK government austerity policies (Byrom, 2013). Within Progression Programme activities described above, legal support, coaching and training was added. The project aimed to build legal capability amongst peer advocates, and support peer advocates to overcome their own legal issues. This included Progression advisors working with peer advocates to discuss legal issues, collate evidence, engage with official bodies and attend meetings like tribunals, and where necessary included a budget for engaging legal support from solicitors. All Groundswell peer advocates were able to receive legal capability training delivered by a specialist third‐sector partner. A second goal in this pilot programme was to extend legal support to be part of the Homeless Health Peer Advocacy Service. Peer advocates were trained to assist clients of the Homeless Health Peer Advocacy Service with identifying legal need and then link clients into support from the Progression Programme. This was a new direction for Groundswell where previously support from the peer advocacy service had been boundaried to address health needs only. We implemented a formative evaluation to guide the on‐going development of Groundswell's services and to add to evidence in this little understood area.

## 2 METHODS

Our design involved a combination of qualitative methods informed by principles of participatory action research and rapid assessment (Israel et al., 2013; Rhodes et al., 2000). We established an academic‐community partnership with Groundswell policy managers, those with experience of homelessness and outside researchers contributing to question development, study design, data collection, analyses and dissemination of findings; participation by those involved in delivering services was central to all five stages, whilst people with experience of homelessness were involved in the first three through a specific research team member, who was then unavailable to join in analysis and dissemination. This central emphasis on participation is a result of an effort to make research more relevant and impactful, as expressly set out in the overall ethos of Groundswell. Action research seeks to explore questions, and through reflection use this knowledge to shape ongoing action (Wallerstein & Duran, 2018). Our approach focused on an open and equal relationship between researchers and participants, to the extent that such boundaries were largely removed in some cases (as evidenced in the study authorship), and of seeking to directly shape practice (Green and Thorogood, 2014). Such action research approaches can extend to a cyclical design, whereby a problem is researched, leading to action and subsequent evaluation, which then prompts further cycles; this had been an original intention, although the complexity of the subject—as we return to in our results—and the dynamism of the institutional context meant this wasn't possible. Rapid assessment emphasises the pragmatic combination of methods with an orientation to providing adequate knowledge to inform policy development (Rhodes et al., 1999). Our orientation was therefore to develop knowledge with immediate relevance for the Groundswell programme, and for this to be part of an ongoing process through the study of iterating between data collection, analysis and considering implications for service delivery. Our core research team therefore included an ‘external’ academic researcher, with long‐standing connection with Groundswell (AG), an ‘internal researcher’ to Groundswell but separate to the actual implementation of the service (MB), and an ‘internal researcher and practitioner’ directly involved as a progression programme manager in working with peer advocates and clients in addressing legal need (AM).

We combined different qualitative methods (Moffatt et al., 2004) to respond to the varying opportunities for data collection afforded by the intervention implementation, and through this foster depth of insight and allow triangulation of findings. We used a series of qualitative data collection methods through 2018: (i) interviews with peer advocates and former volunteers with Groundswell, who both received legal support and engaging with clients with legal need—both peer advocates and volunteers have experience of homelessness themselves, and throughout their work with Groundswell may experience recurrence of homelessness, or be involved in ongoing efforts to respond to issues raised by their past experiences; (ii) interviews with people who are homeless and so potential clients of legal support, and so here understanding that anyone who is homeless could be a potential client if they are experiencing legal problems, and (iii) stakeholder interviews. These modes of data collection were linked to (iv) regular reflective meetings of the research and service delivery team (varying combinations of AG, MB and AM along with other Groundswell colleagues), including those implementing the legal support, which functioned as group interviews. Owing to the immersion in context of MB and AM we also consider the study to borrow from ethnographic approaches to research; with their role as ‘participants’ in the setting studied integral to the data collected and how it was analysed and interpreted.

Sampling for interviews was purposive, aiming to secure a range of experiences, with a priority of gaining information‐rich accounts. The experiences explored are not intended to be typical of all Groundswell peer advocates, volunteers or clients, but instead of the sorts of experiences the legal work was and could be engaging with; we return to this point in reflecting on the study limitations below. In understanding experiences of homelessness and legal support, we followed the approach of official UK definitions of considering homelessness broadly, to include people rough sleeping, those in hostels and temporary accommodation or those in unsuitable accommodation; such a broad definition corresponds to Groundswell's approach of allowing people to self‐identify as homeless in accessing their services. We did not specify participants having any specific legal need, reflecting the exploratory aims of the study and how potentially all clients of Groundswell were potentially likely to receive future legal support. The numbers of people interviewed were shaped by budget and time constraints, and this was within the parameters of a rapid, participatory evaluation. Interviewees were approached either in the context of ongoing work within or by Groundswell, or by direct approaches based on known contact details.

Semi‐structured interviews with peer advocates and volunteers were used to explore legal support needs, the potential and then actual experience of legal support, and how they provided legal support to clients. Topic guides were developed through discussion across the research team. Interviews were conducted by a peer researcher from Groundswell staff or AG. Interviews were done within Groundswell's office, audio‐recorded and lasted approximately 45 min to an hour. A small number of semi‐structured interviews were done with clients of the Homeless Health Peer Advocacy service to triangulate with, and contextualise, the accounts from peers and Groundswell staff; we did not seek a high number of these interviews for the challenges involved in implementing them, not least the burden it places on people who are homeless to recount their experiences (itself potentially a lengthy and complex process, as we detail below in the results). The interviews were used to explore legal support needs, the potential and then actual experience of the intervention. Interviews were done within Groundswell's office and in community settings, audio‐recorded and lasted approximately 45 min to an hour. We interviewed stakeholders involved in the delivery of services to people who are homeless in order to gain an external perspective on legal support. Interviews were structured, brief and held in a space convenient for the interviewee (usually their office). In the group reflective meetings the research and service delivery team would explore specific experiences of those implementing the legal support and of those receiving it, building on the interview data and participants own experiences; we would also reflect on emerging themes from data collection. These meetings were audio‐recorded and transcribed, and so function as data, as well as being integral to our analysis (i.e. it is where initial ideas and analytical and interpretative thoughts were shared and explored).

We interviewed four current and four former peer advocates or volunteers from Groundswell. We also interviewed three stakeholders, who had various positions within the London homelessness response and worked across NGOs, and also three current clients of Groundswell who had received legal support or might be a future beneficiary (more demographic data or background information on experiences of homelessness or legal challenges is withheld to protect the identity of participants). Finally, the Groundswell staff and research team engaged in seven recorded reflective group meetings where specific experiences of peers and clients were discussed and particular analytical themes explored.

We followed a team‐based approach to analysis. Analysis and interpretation of data were shaped by the ‘insider’ immersion in context of MB and AM alongside a sociological ‘outsider’ perspective of AG. Emerging themes in data collection were the basis for ongoing reflection in reflective meetings. After data collection had finished we transcribed all data and conducted a thematic analysis (Green & Thorogood, 2014). Initial open coding was led by MB with assistance from AG, responding to themes and codes identified through the group discussions. In parallel, an internal report (led by MB) and draft manuscript (led by AG) were prepared where initial thematic analyses could be presented and discussed. Reflection on these drafts fed back into the ongoing coding and development of themes. Initial findings were also presented at a conference. Later, AG coded the data drawing on the framework of the themes developed so far, to refine and further develop the themes generated through the discussion and interpretation so far. This manuscript was again discussed repeatedly amongst the team.

Data collection and analysis were shaped by the institutional positions and identities of the researchers. The interviews with peer advocates and clients were led by researchers already known to the respondents through their work in delivering services and in conducting research, which may have limited some aspects of accounts, but also provided a trusting foundation for in‐depth exploration. The role of MB and AM as ‘insider’ researchers also generates partial perspectives, whilst bringing unique insights not accessible to ‘outside’ researchers (Surey et al., 2021). All data were collected by male researchers.

The study had formal ethical approval from ‘King’s College London (ref RESCM‐18/19‐6522). We avoid use of demographic information and detail of legal issues and experiences owing to potential for indirect identification of individuals.

## 3 FINDINGS

We report three core themes: an experience of overwhelming complexity in relation to legal need, how Groundswell peer advocates gained legal support in a context of moral support, and then the challenges in supporting clients.

### 3.1 The experience of overwhelming complexity of homelessness and legal problems

Whether in respondents describing their own experiences, during stakeholder interviews or in team debriefs, there was a recurring theme of the complexity of cases that peer advocates, and volunteers and Homeless Health Peer Advocacy clients face, resulting from specific issues that were understood as hard to resolve, overlapping issues, the lengths of time these legal issues have existed, and then the network of services that they have engaged with. One peer in describing their immigration issues says ‘it has become so complicated’, with ‘deadlock’, and they ‘feel finished’ after their appeal rights are ‘exhausted’, and still there is ‘another insurmountable challenge’ as they try to find another solicitor. Another client describes interlinked problems they face of benefits being withheld, which then creates rent arrears, and this in the context of long‐running mental health problems, with this complexity undermining their own knowledge of what they were receiving:

I don't even know what benefit I'm on now, seriously, I don't know. I did get sick money but that stopped and I was going ESA [employment and support allowance] and I had to go for mandatory appointments with doctors. I missed a couple of them and I had to go for appeal […] I got vouchers for the food bank, [unclear] I got hassled with the housing because I hadn't paid, and my rent arrears went up.

This complexity extended to being challenging to understand for the professionals involved, within Groundswell and outside:

This is one of the challenges that we see with some of the people we are working with. The complexity of their issues … takes us 6 months to get to the bottom of what's going on … and certainly by that point we understand that it's an immigration issue. Or its primarily a criminal justice issue, or its primarily its … this is what is driving everything. (Stakeholder)

This complexity we characterise as reflecting system properties, rather than of the individuals. Complexity was linked to overlapping systems, limits on support being available, and also being difficult to access when it was available, which could include financial barriers of paying for transport to attend appointments, or difficulties in evidencing legal need. People often had long‐running engagement with various interlinked housing, legal, benefit and/or immigration systems. As articulated by a Groundswell progression manager: “everybody that comes through seems to have … seems to already be well connected to services and just need the kind of support that doesn't exist. Or … [support] does exist but they are not able to engage.”

This systemic complexity generates particular experiences of feeling overwhelmed. The perceived process, the toll it might have on the individual's wellbeing and the possibility of a negative outcome were often represented as too much to tackle:

I think that is how everyone that we deal with feels, totally overwhelmed. Whether it's with any direct legal stuff, whether it's with their housing, their benefits. Anything. Job centre. Anything. They just feel I can't deal with this.—Groundswell progression adviser

Other data point to other experiences that potentially result from this complexity. For example, peer advocates and clients sometimes used language such as lacking ‘motivation’ to engage in legal challenges. Here a peer advocate explores their response to being illegally evicted:

I hadn't got the motivation … it's one of those things that … if I don't know about something, I won't … I just can't be arsed sometimes. It's finding the motivation. If I know I have got somebody who can do it for me, then let them … you know. I know that sounds er … but it's true.—Groundswell peer advocate

Language of ‘motivation’ suggests individual responsibility for limited progress on legal challenges; however, in light of the system's complexity, we describe and interpret experiences like this as potentially reflecting an experience of being overwhelmed by complexity.

### 3.2 Legal support to peer advocates and volunteers enabled by a supportive organisational culture

Framed by an experience of complexity, peer advocates, Groundswell staff and stakeholders described legal support as important for peer advocates. They described various benefits or impacts: enhanced knowledge of the law, confidence to address issues, reduced anxiety and improved health.

A central process was signposting to outside support and brokering that support from others. Progression advisers play an ‘Information, Advice and Guidance’ (IAG) role: specialist instruction on navigating support and steps to manage an issue. Another way peers described this support was around ‘letter writing’ and the composition of information. Composing and presenting information in a formal way can be a core step to address identified issues:

First of all he [progression adviser] listened to my needs, he listened to what I was saying. And I was about to get thrown out of my flat so … so he wrote me a support letter, he talked me through the whole process of what would happen.—Groundswell peer advocate

Letter writing and providing information ‘translates’ formal and bureaucratic language from statutory authorities, and makes it accessible, in both directions:

He was helping me write letters. Writing letters for my appeal. I would write a letter draft it, he would read over it. And offer some suggestions. I think I was quite angry during the time, so he was trying to make me soften my language. Or not come across as a prick. Or like … just … er … not too self‐righteous. Which is really helpful. (former volunteer)

By breaking down this language, and then collating and composing information back into a language typically used in legal settings, the progression advisers support peers to successfully engage with state institutions. A peer advocate describes the support they received in processing necessary information and documents:

At the moment I am working on a big case with [progression adviser] regarding my home. And … there is no way—I just came to a point when I said you know what, I am giving up. And she said no, so we finished. Now we have all the documents and everything ready. I slept … honestly for the first time. I am really happy. Because she was there and she was … regularly saying [name] look, we will print the statement, we will fight it like that, and we will annexe it. And I couldn't have done that.—Groundswell peer advocate

Another central role was in brokering support from other agencies, including motivating services already engaged in an individual's case and engaging new support, whether in talking to solicitors, job centres or other charities. A Groundswell advisor illustrates how they pull together and build on support clients are receiving from relevant organisations:

We have got someone who has got issues with welfare and rent and things. So he has got a support within the housing association or the council. We are having a three‐way meeting to ensure that he gets the support. So collaborative working, I think, is another way that we can just … not just on legal support, but on other aspects of support. If that makes sense. So if you think about … making sure … trying to link people into the support that is already in place or the services that are already there.—Groundswell progression adviser

As above, people's experiences are often complex and born of complex systems. Responses indicated that to understand those experiences, and respond in an appropriate way, is perhaps only possible to do in a setting where there is trust and where the handling of an issue can unfold over time:

I mean one of the great things about Groundswell is that there is a welcoming atmosphere and environment. So people do immediately feel comfortable. And it affords enormous trust. And the thing that comes with the trust is disclosure. So people will tell you what is going wrong as much as they will tell you what is going right.—(Groundswell staff)

The provision of legal knowledge or sign‐posting to another form of support we see as embedded in or enabled by an organisational culture of seeking to welcome and accept people, and provide moral support in confronting challenges, drawing on principles of person centred care—understood by Groundswell to emphasise relationships and aiming to respond to an individual's needs and circumstances —and motivational interviewing. Peers described ‘compassion’, having ‘security’, being treated as ‘family’ and that it was like having ‘someone to go into bat on your side’. This role for a particular organisational approach and culture is elaborated here:

I don't know, it's more like … all friends together. It's not as though I have come to seek advice and they are giving it me. We sort of sit down and this is where we have known each other for years. Its … friends meeting for a coffee in a café. It's like that. There is no sort of formula to it. (peer advocate)

### 3.3 The limited potential for legal support to clients from peer advocates and volunteers

Accounts from peer advocates, clients and Groundswell staff emphasised there was some potential to support clients and for this to be mediated by the peers, but how ultimately this was limited and faced significant challenges.

Experiences were described involving positive outcomes from legal support to clients. For example, Peer advocates discussed how they were able to offer their own examples of overcoming barriers as inspiration and signpost the clients they were working with to support services:

In [day centre], I was just having lunch and one of the workers said to him he [the peer] is [same nationality] go and talk to him. He came and talked to me and he says … I have a problem. And he has the same problem as me. Exactly the same problem. And I had [London borough] Law Centre papers in my bag. So I said hey, here is the phone number, call them, its free of charge and they can help you. And god bless, he is working with the [London Borough] Law Centre.

Support from the progression managers was also described by clients in ways indicating it made a substantial difference to addressing their needs.

Challenges to legal support to clients were though raised. One challenge was managing the boundaries of the work, in terms of managing relationships with other organisations and ensuring Groundswell work effectively. The expansion from the focus on health to legal support creates challenges for coordinating work with partner organisations:

this is a tricky boundary issue which people face, in my experience with Groundswell, numerous times before. Where services, service providers get confused about what it is that we offer … so services that we have a strong relationship, with, good relationship […] know for a long time what we do. […] So … there is a … there is a level of symbiosis. Health relationship. But I think the tricky thing is how Groundswell as an organisation supports peers and case workers to maintain that boundary and add value in relation to specifically knowledge of welfare benefits, knowledge of law.—Groundswell progression adviser

Whilst systemic challenges of coordinating with other agencies around clients' cases were perhaps possible to address, more difficult was how the complexity of clients cases related to the particular institutional context of Groundswell. Clients are often still in unstable situations, in some cases still street homeless and or in other points of personal crisis particularly with their mental health. In this context obtaining the necessary information and trust from clients to successfully address legal need could be challenging. One peer advocate explains working with someone street homeless:

Well … obviously the … information, you know, came from dialogue with him. Initially. You know, he was talking about I don't even know where the probation is. He was totally confused. He was … mentioning what was seemingly a serious issue one day and then two days later [unclear] you know … his communication was … er … you know the way he communicated his needs and the issues … going back and thinking about it was very … bitty. It wasn't. You know, it's not like you sit down with him and he goes these are the areas in which … You know. He was coming out as he thought about it, you know. He was just managing his life as it was. So it came up—things came up bit by bit over a period of time.

Whilst experiences such as this could be addressed by the peer advocates and the Groundswell team, their complexity has significant impact on the time of staff, and so potentially limiting support that can be given elsewhere. As above, the work between progression advisors and peer advocates unfolded over time and within the context of on‐going contact within a specific organisational setting; this setting we see as allowing for some mitigation of systemic complexity. Such ongoing engagement and connection is not always as possible with clients, who are more likely in contact with Groundswell for shorter periods of time.

## 4 DISCUSSION

This participatory evaluation of Groundswell's integration of legal support for both peer advocates and volunteers working in the service, and then also service clients, found three core themes. First, Groundswell peer advocates and volunteers as well as clients have an experience of being overwhelmed by complexity, grounded in the multi‐faceted and long‐term nature of legal need and the systems that create and govern responses to legal need. Second, the legal support to peer advocates and volunteers aided in brokering and signposting to legal support, and this was enabled through a particular organisational culture. Third, the provision of support to clients was understood as highly challenging, even if the trust and relationships peers have with clients could allow for some support.

These findings indicate specific directions for future development of Groundswell programmes, and for other third‐sector organisations in the UK and those in similar settings. The results suggest provision of legal support to peer advocates, and so potentially other volunteers and people with lived experience of homelessness working in services, is feasible and could have considerable impact. Our findings suggest the organisational culture of a service is essential. Since the pilot was delivered Groundswell has grown to have a UK wide presence and has further developed its progression programme to be more focused towards supporting Peer Advocates and staff with experience of homelessness to progress into fulfilling careers. Central to this strategy has been further employing psychologically informed approaches to build wellbeing whilst addressing legal and bureaucratic needs. Other similar third‐sector organisations could adopt these approaches. This conclusion comes in the context of increasing recognition of the important role for people with lived experience of homelessness in supporting health goals (Barker & Maguire, 2017), but that specific support is essential to enable this role (Miler et al., 2020). Our findings add to an understanding of how to enable successful peer advocacy programmes.

The provision of legal support by third‐sector organisations like Groundswell to clients currently homeless is challenging. The number of challenges (Fitzpatrick et al., 2011) and system complexity people face (Thompson et al., 2020) and the organisational complexity needed to address this, without the benefit of long‐term relationships, make support to responding to legal issues potentially difficult for a third‐sector health‐focused organisation like Groundswell facing resource constraints. Responses to this could include greater resourcing for third‐sector organisations like Groundswell and also to organisations that have sustained contact with clients.

Recognising the severe challenges for the third sector in responding to legal needs of people currently homeless raises questions over what other sources of support could be available. The complexity of legal need identified, and the experience of this, alerts us to the overall environment of London and the UK producing these experiences. Principal challenges include experiences or fears of hostility from health care (Harris, 2020), policing and the criminal justice system (Moran & Atheron, 2019), immigration authorities and welfare (Thompson et al., 2020). Recognising the role for government agencies in producing the complexity of legal need has implications for identifying sources of legal support, and the feasibility and desirability of third‐sector organisations integrating with government efforts. Such challenges are illustrated in recent tensions over UK charities aligning with government immigration policies (Taylor, 2019). Integration of third sector and government agencies therefore requires caution.

Previous experiences have shown the potential for welfare advice within primary health care settings (Moffatt et al., 2004); a benefit of this for how institutional cultures and priorities in health care settings can be distinct to that of other areas of government (Berlin et al., 2019), although we note how delivery of health care in the UK has a long history of policing immigration (Nasar, 2021). Whilst needing to address those concerns, particular initiatives include specialist case workers within specialist primary care for people who are homeless, who can provide support on legal need as well as housing and health (Low Commission, 2015). Such programmes could be an alternative source of support to, but also mediated by, peer advocacy, for example through peer advocacy working to enable links and relationships with these specialist case workers, in acknowledgement of the broader skills and role for peer advocacy in bridging the multiple gaps to care and support for people who are homeless (Barker & Maguire, 2017; Finlayson et al., 2016).

These results need to be understood within the limits of a small participatory action research study. Resources and time were limited, and our priority was to provide practical insight to an emerging service. The findings here have specific implications for Groundswell, and considering the limited data we draw on any findings should be considered cautiously by other similar organisations implementing or planning similar work. We also note the potential for desirability bias throughout our interviews, for how many respondents were in a position of ongoing support from the organisation leading the study; our analysis has allowed for this, and sought to develop critical insight, including through the process of participatory reflection. That the study methodology incorporates principles of participation and action research with those delivering an intervention integral to its evaluation also brings potential for bias; whilst so, this inclusion of insider perspectives is also a strength, through bringing insight to institutional processes normally hidden to outside researchers. Further research to research by others to explore and add to our initial findings.

In conclusion, our participatory action‐research of Groundswell's legal support suggests this service can help in addressing the system complexity facing peer advocates. Novel services such as the delivery of legal support by health‐focused organisations do though bring risks, and in consequence of these, legal support to clients currently homeless is currently highly challenging. Cross‐sector debate in the UK is needed to identify further opportunities to address the complexity of legal need facing many people who are homeless which ultimately complicates health.

## FUNDING INFORMATION

The evaluation and service evaluation were funded by the Legal Education Foundation.

## CONFLICT OF INTEREST

MB and AM were both working for Groundswell, who were funded by the Legal Education Foundation to implement the pilot programme being evaluated. AG declares no conflict of interest.

## ETHICS STATEMENT

The study had formal ethical approval from King's College London (ref RESCM‐18/19‐6522).

## Data Availability

Research data are not shared owing to the risk of indirect identification.
